# Psychological distress in parents of children treated for cancer: An explorative study

**DOI:** 10.1371/journal.pone.0218860

**Published:** 2019-06-21

**Authors:** Tommy Carlsson, Laura Kukkola, Lisa Ljungman, Emma Hovén, Louise von Essen

**Affiliations:** 1 Clinical Psychology in Healthcare, Department of Women’s and Children’s Health, Uppsala University, Uppsala, Sweden; 2 Sophiahemmet University, Stockholm, Sweden; 3 Department of Women’s and Children’s Health, Karolinska Institutet, Stockholm, Sweden; University Medical Center Utrecht, NETHERLANDS

## Abstract

**Objective:**

To explore psychological distress experienced by parents who express a need for psychotherapy after curative treatment for their child’s cancer.

**Methods:**

15 parents (eight mothers and seven fathers) of children treated for cancer (median time since end of curative treatment: two years) were recruited via a pediatric oncology center. Each parent was interviewed twice and data was analyzed with inductive latent qualitative content analysis.

**Results:**

Two overarching themes emerged. One theme, *An unfamiliar and frightening situation during treatment*, portrayed experiences during the treatment period, and included the sub-themes *Initial reactions to the uncontrollable situation*, *Adjustment to the situation*, and *Focus on supporting the child*. Another theme, *Emotional struggles after end of curative treatment*, portrayed experiences following curative treatment, and included the sub-themes *Transitioning back to life as it was before the diagnosis*, *Emotional scars*, *Uncontrollable fears and worries of diseases*, and *New perspectives on life*.

**Conclusions:**

Parents of children with cancer experience existential, physical, psychological, and social struggles. They describe an unstable situation after diagnosis and having focused their attention towards protecting their child during treatment. After the end of curative treatment, they experience challenges with transitioning back to life as it was before the diagnosis and dealing with their own emotional scars and fears related to the child’s cancer. The findings indicate an unmet need for psychological support among parents of children treated for cancer.

## Introduction

The survival rate for childhood cancer has increased dramatically, and is now approaching 80% [1]. Thus, most children diagnosed with cancer experience the end of curative treatment and transition into survivorship, a period characterized by unique challenges for both survivors and their parents. A high proportion of parents report negative psychological effects in connection with the diagnosis, including symptoms of post-traumatic stress. For most parents the psychological distress declines during the initial months following the diagnosis, thereafter the decline abates and from three months after end of treatment only a minimal decline occurs [2]. Indeed, research shows that a substantial subgroup continue to report a high level of anxiety, depression, general psychological distress, and/or post-traumatic stress symptoms (PTSS) up to 10 years after the child’s diagnosis [2–6]. Psychological distress *per se* is associated with low quality of life, functional disability, and an increased risk for somatic disorders [7,8]. Symptoms of post-traumatic stress are known psychological consequences among parents of children diagnosed with cancer, with clinical challenges related to screening routines and psychological treatment [3,5]. PTSS interferes with parents’ cognitive processes and executive functioning [9], potentially hampering their capability to participate in important decision-making about their child’s treatment and provide emotional support to family members. For some, these reactions can develop into post-traumatic stress disorder (PTSD), a persistent and distressing syndrome.

Even though most parents recover from the distress that they experience during the time of the child’s illness and treatment, a substantial subgroup reports a need for psychological support after end of curative treatment. Only limited research has focused explicitly on the nature of distress in this subgroup of parents. This represents a significant gap in the literature given that only a subgroup of those who report a need for psychological support after the end of curative treatment receive support whereas most who receive support find it beneficial [10]. Psychosocial care and psychotherapy have the potential to benefit these parents. However, research indicates psychological support is not routinely offered to parents in Sweden [2,10]. This indicates that the needs of psychological support among parents of children treated for cancer are not met by current Swedish standard care. Acceptable, relevant, and clinically effective psychological support for the population should be developed according to the population’s experienced distress and needs. More explorative research is needed to achieve a wider understanding of parental experiences within as well as beyond the established diagnostic frameworks for psychological distress. With the overarching purpose to provide insights concerning how to develop acceptable, relevant, and clinically effective psychological support for parents of children treated for cancer, the aim of this study was to explore psychological distress experienced by parents who express a need for psychotherapy after curative treatment for their child’s cancer.

## Method

### Participants

Parents were eligible if they had a child who, by the time of consideration, had completed successful cancer treatment at the pediatric oncology center at the Children’s University Hospital in Uppsala, three months to five years earlier. They needed to be able to speak Swedish and commute to either the hospital located in Uppsala or Västerås, where the interviews were conducted. Moreover, they needed to confirm that they experienced psychological distress of any kind, which they related to their child’s cancer, and expressed a need for psychotherapy. Parents were excluded if they suffered from a psychiatric disorder in immediate need of treatment (e.g., suicidal ideation) or if they were undergoing psychotherapy. Assessment of severe psychiatric comorbidity was based on a clinical judgment of participants’ ratings on the Montgomery–Åsberg Depression Rating Scale (MADRS-S) [11,12], and the diagnostic interview Mini-International Neuropsychiatric Interview (M.I.N.I.) [13].

Recruitment started 02/01/2013 and ended 02/15/2014 during which 80 potential participants were identified and assessed for eligibility. The potential participants were identified by staff at the pediatric oncology center at the Children’s University Hospital in Uppsala. Parents whose children had completed successful cancer treatment at the pediatric oncology center three months to five years earlier were identified through hospital records and contacted by health professionals at the center. The healthcare professionals provided brief information about the study, and compiled a list of all parents who expressed an interest in participating and orally consented to receive more information. One of the psychologists working with the project systematically contacted all the parents in the list provided by the hospital via telephone. During the phone call, the psychologist provided more information about the study, addressed any questions that the parent had about the study, and confirmed eligibility according to the inclusion and exclusion criteria. Of the 80 potential participants, 48 (60%) were excluded, as they did not report psychological distress related to the child’s cancer. Of the remaining potential participants, 17 (53%) declined participation, the most common reason being that they did not want to be reminded about the child’s cancer. The final sample consisted of 15 parents (mothers n = 8, fathers n = 7).

### Data collection

The overall purpose of the interviews was to identify the participants’ thoughts and feelings related to having a child previously treated for cancer. To generate rich data, open-ended interviews were conducted twice for each participant, and lasted until the respondent felt like they had no further information to share. The first interviews began with the question “Could you please tell me about your thoughts and concerns related to your child’s cancer?”. The second interviews were conducted to encourage the participants to take some time to reflect on what was discussed earlier, gather preliminary findings, and have the possibility to return and expand upon topics that were insufficiently covered in the first interview. Thus, our intention was that the second interview would aid in the process of exploring the parent’s experiences in as much detail as possible. The second interviews were performed with all participants. The time frame between the first and second interview was approximately one week. The participants did not participate in any intervention during this period. Probing and follow-up questions were asked, according to the topics brought up. Examples of probes and follow-up questions used include “could you please tell me more” and “can you describe that in more detail”.

The interviews took place in a private room at the hospital in Uppsala or Västerås respectively, and lasted approximately one hour each. Participants were not reimbursed for their participation in the study. One participant did not speak Swedish, and was thus interviewed with the help of a professional interpreter. Data collection continued until the interviewers concluded that no new experiences were described when the second interviews were conducted. The interviews were audio-recorded and transcribed verbatim by a professional transcription agency. Two psychologists (including the third author) with formal training in cognitive behavior therapy and no relationships with the participants prior to the commencement of the study conducted the interviews. If both parents of the same child were included in the study, different psychologists interviewed them.

### Data analysis

The transcripts were analyzed with inductive latent qualitative content analysis, a method with the purpose to describe overarching patterns found in text-based data. In latent analysis, the focus is directed towards a high degree of interpretation of the underlying meaning, identified through a deeper level of abstraction [14]. The analysis was inspired by the outline presented by Graneheim and Lundman [15], and followed an inductive approach involving the following steps: (1) each transcript was read repeatedly to gain an overall understanding of its context and content; (2) meaning units were identified, defined as sections that shared a common meaning and context related to a psychological reaction; and (3) meaning units were assigned a descriptive code, defined as a label of the meaning unit’s content. If specified in the interview, codes were labeled according to whether the content was related to the time during or after end of the child’s curative cancer treatment. Codes were sorted into sub-themes and themes, defined as overarching threads that refer to an interpreted underlying meaning of the collected data. Because of the intertwined nature of human experiences [15], themes were not considered externally heterogeneous. Table 1 presents examples of the analytic process.

**Table 1:** Examples of the analytic process.

Meaning unit	Descriptive code	Sub-theme	Theme
At that time, you didn’t think about yourself at all, you only thought about how it all would turn out for [the child].	Did not think about themselves, only the child [during treatment]	Focus on supporting the child	An unfamiliar and frightening situation during treatment
At the time of his disease, when he was in treatment … Yes, at that time it was more like, well, the focus was on [the child].	The focus was on the child [during treatment]		
You learned to find the good moments in everyday life. Because everything didn’t feel dark during [year of treatment]. We had moments, or small things that we did when [my child] had the energy.	Found good moments in everyday life [during treatment]	Adjustment to the situation	
You wanted to be there for the other two also, we have three kids so it is difficult to… find this balance when it is fair and not just fair but also that they would feel that they have a mommy who cares about them, even though she [the ill child] is sick.	Want to be there for the other children also, find a balance in what is fair and that they feel that they have a mother who cares [during treatment]		

The first and second authors separately analyzed one interview. They then reviewed their identified meaning units and codes for this transcript, with the purpose to explore their analytic approach and identify differences with regard to their interpretations. The review was made during face-to-face meetings were the two authors jointly scrutinized all the identified meaning units and codes. The process revealed that the analyses were highly similar. Thereafter, the first author analyzed 24 of the remaining interviews, whereas the second author analyzed five. The first and second authors held repeated meetings during the course of analysis, where they discussed the identified meaning units, codes, and overarching sub-themes and themes. The purpose of using two analysts was to gain a deeper and nuanced understanding of the parents’ experiences. When all interviews were analyzed, the remaining authors were invited to review the transcripts and share their reflections until they all considered the interviews to be thoroughly covered. At this point, all remaining authors judged that the findings thoroughly portrayed the data. Thus, no changes were made with regard to the findings after their reviews. The thematization of codes was managed in Nvivo.

### Ethical considerations

The authors assert that all study procedures comply with the ethical standards of the relevant national and institutional committees on human experimentation. The procedures were approved by the regional ethical vetting board of Uppsala (approval number: 2012/440) and all participants provided written informed consent.

## Results

### Sample characteristics

All participants except one mother were in a relationship. The sample was diverse with regard to background characteristics (Tables 2 and 3). One child had received a second cancer diagnosis >10 years after primary diagnosis and one child had a history of relapse.

**Table 2:** Background characteristics of mothers (n = 8) and fathers (n = 7).

Variable		Mothers	Fathers	Total
		n	n	n
Child’s cancer diagnosis	Brain tumor	1	1	2
	Wilms’ tumor	1	-	1
	Bladder tumor	1	-	1
	Germinoma	1	-	1
	Ovarian cancer	-	1	1
	Acute lymphoblastic leukemia	-	1	1
	Acute myeloid leukemia	1	-	1
	Hodgkin’s lymphoma	-	1	1
	Neuroblastoma	1	1	2
	Rhabdomyosarcoma	1	1	2
	Lymphoma	1	1	2
Marital status	Married/partnership	7	7	14
	Single	1	-	1
Living with the child’s biological parent	Yes	6	6	12
	No	2	1	3
Educational level	Below university	4	5	9
	University	4	2	6
Employment status	Employed	5	4	9
	Unemployed	1	2	3
	Sick-leave	2	1	3
Sex of child	Female	3	4	7
	Male	5	3	8

**Table 3:** Ages of mothers (n = 8) and fathers (n = 7), their child’s age, and time since the end of curative treatment for their child.

Variable	Mothers		Fathers		Total	
	Median	Range	Median	Range	Median	Range
Parents’ age at data collection (years)	42	35–50	45	36–52	44	35–52
Children’s age at the time of diagnosis (years)^1^	9	1–14	8	1–14	8	1–14
Children’s age at data collection (years)	13	3–20	12	3–21	12	3–21
Time since end of children’s curative treatment (years)	2	<1–5	2	<1–4	2	<1–5

^1^ Age at primary diagnosis.

The median age of the participants was 44 years (range = 35–52), and the median age of their children was 12 years (range = 3–21). The median age of the children at the time of diagnosis was 10 years (range = 1–15), and the median time since the end of curative treatment was two years (range = <1–5 years).

### Qualitative results

The analysis resulted in two overarching themes: *An unfamiliar and frightening situation during treatment* and *Emotional struggles after the end of curative treatment*.

#### An unfamiliar and frightening situation during treatment

The theme concerns experiences during treatment and includes the sub-themes: *Initial reactions to the uncontrollable situation*, *Adjustment to the situation*, and *Focus on supporting the child*.

**Initial reactions to the uncontrollable situation.** The time of diagnosis was described as chaotic, involving unexpected and uncontrollable emotional distress. However, relapse was not associated with the same emotional distress as the initial diagnosis. The treatment period had been emotionally and physically exhausting, including sleeplessness, eating difficulties, and nightmares. The situation was experienced as deeply unfair, and uncontrollable anger towards others who were perceived as ignorant and careless was experienced.

I thought many times—especially when I stood by the window and watched people walking outside—that there you are, and here I am in this bubble. And you [people outside] have no idea what I go through in here. You don’t care, you go on and live your lives. And I could get really angry about that.

(Mother 1)

However, when a relapse was diagnosed, this was not associated with the same emotional distress as the initial diagnosis.

I think we were more worried the first time when she was little, that we thought that now we have to stay away from everything and everyone, but as I said… You learned that rather quickly. So it wasn’t even a surprise when she got ill the second time, in some way we knew quite well what would happen then so… I didn’t find that as distressing [as the first diagnosis].

(Mother 1)

Thoughts about death and mortality were experienced, particularly fears of losing the child. Religious and fatalistic thoughts were also described, such as questioning why God had given them a child who may die young. Respondents tried to actively avoid these thoughts, and became emotionally distressed when confronted with these thoughts. Occasionally, their children talked about the risk of dying, which had been stressful and difficult to deal with.

So, I thought: “Why does God give me a child that I only get to have for three years?” […] at that time, I had already started to plan how I would arrange his funeral.

(Mother 1)

**Adjustment to the situation.** Following the diagnosis, respondents needed to adjust to the new situation with a hospitalized child. At times, successful adjustment was experienced, during which the hospital became a part of life and a place that symbolized safety. Occasional moments of joy and appreciation were experienced at the hospital, including the opportunity to spend time with their child.

You learned to find the good moments in everyday life. Because everything didn’t feel dark during [year of treatment]. We had moments, or small things that we did when [my child] had the energy. And there is a nice assortment of fun things to do with your child [at the hospital] that the Swedish Childhood Cancer Fundation arranges. I mean, we completed every single puzzle at the ward and noted which pieces were missing and which puzzles were complete. We had a blast when we did that. We watched every movie that was available. That was also really fun. And we ate tons of candy, or I did. He watched… and maybe took one bite. But it was cozy. We created our own cozy world in this world.

(Mother 1)

At other times, the hospital stay was described as psychologically draining, with slow and boring days. Admittance to intensive care units were described as particularly chaotic and unaccommodating.

There was noise everywhere, and like, we sat on solid fold-up chairs for several days. So it [the intensive care unit] was not adapted well enough to accomodate any relatives of admitted children.

(Mother 6)

Having a child in treatment meant each parent dealing with their own emotional distress and simultaneously trying to be a supportive parent. Parents described having experienced a need for psychological support and having received helpful and appreciated support from nurses, physicians, and social workers. Considerable trust in the competence of certain healthcare professionals was reported. However, a lack of professional support and not feeling adequately supported was also described. Some healthcare professionals had been difficult to communicate with, and had shown little understanding and interest in their well-being.

They [healthcare professionals] didn’t really have much understanding. We were placed in an orthopedic unit for children and had to share room with another child, and that felt really tough when you have a child that is severely ill due to cancer.

(Mother 4)

Respondents avoided external reminders of the cancer presented to them during the treatment period, including hair loss and administrations of drugs. For some, work served as a welcomed distraction during the treatment and was described as a useful coping strategy. At work, respondents felt free to think about less distressing topics than the child’s cancer for a while and were placed in a position where they were required, in a positive way, to interact with others.

Overall, going to work is a positive thing. Because then you don’t shy away [from people], instead you get to meet people in everyday life.

(Father 2)

Social support was desired and appreciated. However, breaking the news about the child’s cancer and having to answer cancer-related questions from others had been a tough challenge for some. Instead, some described that they chose to isolate themselves from social interactions. Some friends had stopped contacting them after the child’s diagnosis resulting in a lack of social support. However, other friends contacted them frequently and provided both emotional and instrumental support, which was greatly appreciated. Communicating with peers, i.e., relatives of adults or children with current or previous cancer, were described as highly appreciated, since these persons understood their situation in a different way than others who did not share their experiences.

He went through the exact same treatment as [my child], the same medications, the same everything, which made it, like, it was kind of, yes, a bit like therapy. We could talk to each other […] it was pretty positive to have someone to talk to during that period, who had been in the exact same situation.

(Father 1)

**Focus on supporting the child.** Being a parent of a child on treatment for cancer involved a complete focus on supporting the child. In order to do so, respondents tried to keep their own energy and spirit as high as possible and restrained themselves from acknowledging and prioritizing their own psychological well-being. Instead, the needs and limitations of their ill child had been their absolute priority, and a decisive factor in how life at the hospital was experienced.

Even if I felt poor myself, that was never my priority, but rather, my child was my priority. He was the important one, no one else. And, at that time, I did not need to care about myself.

(Mother 1)

Respondents had experienced intense worries and fears related to the child’s safety. To cope with these worries and fears, they had engaged in various behaviors for the purpose of protecting their child from additional suffering. They had constantly striven to stay close to their child, closely observing their child’s physical and mental status, so that they could detect any deteriorations in their health and feel in control. A need to constantly be on guard when their child was cared for by healthcare professionals caused them physical discomfort and psychological distress. For example, they described a great need to continuously monitor them during nights by sleeping next to them, crticially observing and questioning the competence of healthcare professionals, searching for and dealing with great amounts of information, and managing the care of their child.

I took on some kind of role [during the treatment period] as a robot, having complete surveillance over exactly when he needed food, when the gavage feeding was scheduled, when he was supposed to take his medications […] It wasn’t only one thing to keep track of, I had it all under my watchful eye, everything. It was just as if I was a computer that just worked.

(Mother 1)

A need for truthful and correct information from healthcare professionals was also described, related to a desire to feel in control. However, the need to support and protect their child was complicated by experiences of powerlessness and feeling like they were completely in the hands of healthcare professionals. This was particularly stressful, since it contradicted their desire to feel in control of the situation.

We were in the hands of physicians and nurses and that’s… As a parent, you are not informed enough, they know what is best. And from there on, you just have to jump on the train and go along for the ride.

(Mother 8)

Some described feeling torn between supporting the child with cancer and simultaneously trying to attend to the needs of their other children. This resulted in feelings of inadequacy as a parent of their other children who lived at home during the hospital stay. Being torn between staying at the hospital and simultaneously attending to the needs of siblings resulted in feelings of guilt for not being able to take care of all their children.

I wanted to be there for the other two, as well. I have three children and it’s tough to find that balance, so that it is fair [for all my children].

(Mother 5)

#### Emotional struggles after the end of curative treatment

The theme concerns experiences after the end of curative treatment, and includes four sub-themes: *Transitioning back to life as it was before the diagnosis*, *Emotional scars*, *Uncontrollable fears and worries of diseases*, and *New perspectives on life*.

**Transitioning back to life as it was before the diagnosis.** When discharged from the hospital, several parents experienced feelings of having been tossed into a different reality far away from the safety associated with the hospital stay. The contrast between having the child taken care of by healthcare professionals during the treatment at the hospital, and having to take care of their child on their own at home, was described as a stressful transition. Not feeling sufficiently informed about this transition before it took place resulted in a lack of knowledge and feelings of being ill-prepared to manage the situation and take care of their child at home when the child was discharged from the hospital. Following discharge, parents felt that the sole responsibility of taking care of the child was placed on them, and criticized the lack of follow-up routines. The lack of follow-up routines and preparatory information before the discharge from the hospital resulted in parents feeling abandoned during a vulnerable time and worried if their child’s health would deteriorate at home.

The worst part was that we felt like we received very poor support from the hospital at that point in time. When we, like, just left the hospital and went home, well… Then no one really cared about us anymore.

(Father 1)

Following the discharge, respondents wished to get back to life as it was before the diagnosis and to move forward as a family. Some experienced insufficient formal support related to the child’s transition back to school. Parents who experienced a lack of such support tried, through their own efforts, to support their child during this transition. This had been a difficult challenge that required considerable energy and time. Eventually, some started to feel life as it was before the diagnosis return, and that it became easier to plan ordinary activities in their lives. Getting back to work was seen as an important part of getting back to life as it was before the diagnosis. However, great difficulties were described related to trying to balance family life and work.

I keep getting behind in my work all the time; that’s how it is when you can’t work at your full capacity. And then, when I get home, I still have work left sometimes, even though I don’t want to have it like that. It’s a tough balance.

(Father 4)

Following the discharge, relationships with others in their closest social network had changed. In many ways, the relationships with family members had improved and grown closer because of their shared experiences during the treatment period. However, strains in relationships with family members were also described involving arguments, conflicts, and frustrations in life. Impaired relationships with partners, including communication difficulties and not taking part in joint activities were also described.

In the worst moments, I think that we might as well get divorced, because at those times we don’t get any joy out of being together. And at other times I really try to make an effort so that all will get better.

(Father 2)

**Emotional scars.** Emotional scars were experienced years after the end of curative treatment. Sometimes, these overshadowed hardships experienced during the treatment period. When comparing their psychological state during and after treatment, several described it as worse today. Difficulties feeling joy and happiness, as well as a tendency to augment negative emotions, was mentioned. Some experienced intense panic attacks and anxiety, particularly when reminded about previous experiences or in the company of crowds. Respondents described intrusive symptoms when faced with external reminders and experienced flashbacks of distressing scenarios that happened during the treatment. A link between these experiences and insufficient time or energy to deal with their own experiences during treatment was mentioned. Usually, parents began to process their own experiences after the end of the curative treatment, when their child did not require the same amount of attention, compared to during treatment..

You kind of haven’t had time for yourself, I think. During the treatment period, you manage, because you always have something to do, to focus your attention on, something that needs to be done. And there’s no time for anything else. But when it’s over, it just feels empty, you feel weak, you kind of haven’t landed yet.

(Mother 2)

For some, talking about their own psychological health was challenging and stressful. Thoughts about cancer-related experiences caused anxiety and physiological reactions, such as stomachaches and headaches. Sleeping difficulties and nightmares were also described. Respondents described uncontrollable physiological arousal symptoms, unexplainable anger, irritability, and loss of patience towards family members and others were experienced. Respondents who enacted these reactions towards their children expressed a need to change their behavior. Respondents did not know how to deal with these reactions, and felt bad about their own behavior towards family members when struck with uncontrollable anger.

I want to find strategies to help me process everything, so that I don’t have to hear from my children that I yell at them all the time and get angry about everything.

(Mother 1)

Symptoms of avoidance were described by the parents, who actively avoided thinking about experiences and feelings, in particular thoughts related to mortality and death. Consequently, respondents described that they felt most at ease when they were able to forget their own situations. Topics related to their experiences were avoided in conversations with others. The parents wanted to process their thoughts and used various strategies to improve their psychological well-being. Such strategies included attending yoga classes, writing a diary, taking time for personal interests, and taking long walks. Social support was seen as essential to efficiently cope with the situation after the end of curative treatment. Having received beneficial social support from family members, friends, co-workers, and peers was described. However, it was also mentioned that some friends and family members had stopped showing an interest in their wellbeing after the end of curative treatment. For some, this resulted in feelings of loneliness and a percieved lack of social support. Group discussions with peers was suggested by some as a helpful way to deal with psychological distress. However, little or even no need for social support was also described.

It’s wonderful that both my family and my husband’s family are there for us […] it’s great to have someone to lean on. And, on the other hand, I have two families who became the opposite, they are almost terrified to come and see us, I feel that it is regrettable the way that turned out.

(Mother 5)

For several, the period after curative treatment involved symptoms of significant loss of energy and stamina. This had an considerable effect on wellbeing and life, including not having enough energy for household chores or isolating oneself from social contacts. Respondents mentioned that they were unable to bring forth enough energy to do things outside the comfort of their homes. Tiredness and weakness were also experienced, built up over several days and persisted despite time for recovery. Some described that they felt a lack of interest, motivation, capacity, and commitment to participate in leisure activities and do well at work.

When there is something that I will take on by myself, it feels so far away. I think that’s how I notice the most that I feel it’s difficult [to initiate something], because I lose the power to act.

(Mother 3)

**Uncontrollable fears and worries about diseases.** Several described uncontrollable fears and worries, which consumed considerable energy. Fear of cancer recurrence and fear of other diseases in themselves or their family members were experienced, and were stressful. Worries and fears were often evoked when reminded about cancer-related experiences, such as at the time of regular oncology check-ups or when watching television shows about cancer.

The fear is still there, unfortunately. It is. But not in this really, really big way like it was in the beginning, that is, right after the treatment [had been concluded]. Now she has been given a clean bill of health. But still, like, you know that you have it on paper, proof that she is healthy and all that, but the fear is still there.

(Mother 5)

For some, worries and fears occupied much of their time and energy. These parents described themselves as hypochondriacs and hysterics, experiencing exaggregated fears and behaviors that became a nuisance for them in their lives. To cope with the intense fears and worries, measures were frequently taken to prevent diseases and risk of recurrence. Such behaviors included always trying to be extremely clean, constantly being on guard for signs of diseases, and forcing their children to live as healthy a lifestyle as humanly possible. When a child showed any signs of diseases, respondents experienced feelings of panic and horror.

I’m very fearful of diseases, I am very afraid. I can get these manic thoughts and lie and palpate my stomach […] I get angry at my children if they sit and fiddle their noses and eat snot, even though I know all children do. It’s like the logic is there, but my feelings take over.

(Mother 1)

**New perspectives on life.** Having experienced childhood cancer resulted in new perspectives about the fragility of life and a newfound respect of the fact that anyone could die at any moment. Parents experienced an increased value in spending time with family members and a greater concern about the wellbeing of family members. When comparing current priorities to priorities before the diagnosis, the wellbeing of family members was considered a more prioritized aspect today.

I can sit down on the sofa with them [family members], I didn’t do that before, and instead I used to do something else. And I feel like that is great, that I actually take advantage of the moments when I can sit there and cuddle a bit.

(Mother 2)

Changed perspectives on various trivialities and superficial matters in life were described. Such trivialities were not given the same attention and impact as before the diagnosis, since they no longer felt they had enough energy or time to care about such matters. Parents expressed that they no longer complained about trivial things in life. Some also expressed that their work no longer felt joyful or challenging, and thus desired to change their profession. When comparing their current behavior to their behavior before the diagnosis, some described that they now had a tougher attitude towards others in society, with lessened empathy for people who whined about, from their perspectives, banal things.

These things that may be a little shallow in life, we don’t put as much weight on those things any more. Many times, it’s more exciting and worthwhile to talk to a person who has something to offer, so to say.

(Father 2)

## Discussion

This study explored psychological distress experienced by parents who express a need for psychotherapy after curative treatment for their child’s cancer. The results illustrate the multidimensional and temporal aspects related to the psychological distress experienced by these parents. The parents recollected that the treatment period involved a chaotic and uncontrollable initial situation that had been completely unexpected, a need to psychologically adjust to the new situation when having a hospitalized child, and having a complete focus towards supporting their children. After curative treatment, the parents experienced the necessity of transitioning back into a different reality far from the safety associated with the hospital stay, emotional scars presenting and persisting years after the curative treatment, uncontrollable fears and worries that consumed considerable energy, and finding new perspectives on life.

Previous research shows that while most parents are resilient and adapt well following the diagnosis, subgroups continue to report high levels of psychological distress after the end of curative treatment [2,3,5,16]. In accordance with what has been reported in previous studies [17,18], participants felt powerless and experienced a loss of control, and wanted to be offered as much information as possible. Oncology patients regard professional information as an essential aspect of care [19], and poor communication between patients and healthcare professionals is associated with psychological distress and quality of life [20,21]. Parents of children diagnosed with cancer report a considerable need for oral and written information [22], but often experience an unmet need for information [23,24]. Active coping strategies, such as seeking and using patient information, have been associated with less psychological distress [25,26], which further illustrates the importance of adequate information to parents. Taken together, the findings call attention to the acknowledged challenges related to information and psychosocial support in oncology [27], and emphasize the importance of sufficient information to parents following the diagnosis. Additional research is needed among parents of children diagnosed with cancer to describe their satisfaction with professional information and to explore whether there is a potential association between satisfaction with information and long-term psychological outcomes.

The parents described various coping strategies during and after their child’s cancer treatment. Some withheld their own feelings and socially isolated themselves, which may be seen as avoidance or disengagement. Avoidance has been proposed as central to the development and maintenance of psychiatric symptoms in general [28]. In populations exposed to traumatic experiences, avoidance of reminders related to the trauma may increase the level of psychological distress, because thoughts and memories are not confronted and thus not efficiently processed [29]. Findings from studies on parents of children with cancer indicate that early in a child’s illness trajectory, avoidance may be helpful in regulating the amount of information and emotional reactions processed [25]. However, avoidance around the time of diagnosis has been shown to predict higher levels of distress in the long-term [26]. Active coping strategies, such as engaging in work can promote psychological adjustment [26]. Participants in this study described taking on work as an important element in their lives. However, some struggled with balancing the demands of work and attending to the needs of their family members. As also suggested in other studies [6,30], family-friendly work arrangements (e.g., flexible work schedules, remote work) and extended psychosocial support could prove helpful in order to promote successful return to work. There is a need for descriptive and experimental studies that further investigate how to promote return to work, and the possible effects that return to work may have on psychological adjustment.

Various challenges related to the process of transitioning from hospital to home following the discharge were identified. Previous research calls attention to the importance to help parents prepare for this transition [31]. Research shows that parents experience a lack of psychological support from healthcare professionals and social support after curative cancer treatment [10,31]. Although the period following treatment completion represents an opportunity for parents to adapt and improve their psychological health [32], the findings from this and other studies illustrate the presence of continued cancer-related stressors, such as intense fear of recurrence [6]. Taken together, the findings call attention to the psychological fragility among these parents, and further, show the significant impacts that the transition from hospitalization can involve. Judging from the findings, rigorous and systematic screening for psychological distress is of the utmost importance in order to successfully identify parents who are in need for psychotherapy after the conclusion of curative treatment for the child’s cancer.

Most previous studies on the psychological consequences of childhood cancer for parents have used a symptom-focused perspective, often utilizing a PTSD framework [3]. However, the identified diverse manifestations of psychological distress point to a complex symptomatology. Relying solely on one framework may limit the overall understanding of the full impact that a cancer diagnosis may have on parents. Our results suggest that PTSD, depression, and anxiety frameworks are important to take into consideration to fully describe and understand parents’ psychological sequelae. Based on the extensive comorbidity of psychiatric symptoms identified in previous research [16,33] trans-diagnostic approaches to treatment have been attracting increased attention. The parents included in this study described various symptoms that illustrate this, such as panic attacks, sleeping difficulties, substantial energy loss, irritability, physiological arousal, avoidance, intrusive thoughts, negative alterations in mood and cognitions, and diminished interest in leisure activities. Given the overlap of symptoms that parents present, a trans-diagnostic approach targeting processes that underpin different manifestations of psychological distress may be useful and should be given attention in future research.

Positive experiences were also expressed by the parents, which is in line with results of previous studies [34–36]. This illustrates that parents who struggle with psychological sequela and who experience a need for psychotherapy still acknowledge and experience positive aspects. These results suggest the potential presence of posttraumatic growth (PTG), i.e., experienced positive changes that follow challenging life crises. To promote resilience and positive psychological development, positive experiences and the possibility of PTG are important aspects to consider in the development of relevant and effective interventions for formal psychosocial support. However, there is a lack of studies that investigate PTG among parents of children with serious illnesses [5,37]. Thus, more research is needed to fully understand the impact that a cancer diagnosis in a child may have on the psychological health of their parents.

### Methodological considerations

There are some methodological considerations that need to be considered when interpreting the findings. The sample consisted of parents recruited from a limited geographical area in Sweden, only one parent was single, and all but one parent spoke Swedish fluently. We set out to explore perspectives among parents who reported psychological distress, which they related to the child’s cancer, and a need for psychotherapy. Consequently, the results illustrate the lived experiences among a subgroup of parents. We acknowledge that the results need to be interpreted with this in mind, as these are all aspects that have implications on the transferability of the findings. On the other hand, the sample included both mothers and fathers with various backgrounds with regard to their own and their child’s age, the time since diagnosis, educational level, employment status, previous psychological treatment, sex of the child, and cancer diagnosis. It is possible that the child’s cancer was an unacknowledged cause for distress among some of the potential participants who were excluded based on the fact that they did not relate their psychological distress to the child’s cancer. Previous research on focusing effects describe that individuals who are cued to a certain factor before reflecting on their mental state tend to exaggerate the importance of that factor [38,39]. Thus, the number of parents excluded for this reason might be limited. The open-ended interviews allowed parents to share what felt most important to them and produced rich material, which was confirmed by data saturation judged by the interviewers. Data was collected with two interviews, which made it possible to return to previous topics insufficiently covered during the first interview. Participants were interviewed a second time because we wanted to explore their experiences as thoroughly as possible. The second interviews produced rich data that complemented the first interviews and enhanced the exploration further. However, we cannot dismiss the possibility that parents may have chosen not to share certain experiences during the interviews. Two analysts collaborated during the analytic process, one a psychologist and one a researcher nurse-midwife with previous experience of conducting qualitative content analysis. This allowed for two different perspectives on the material. A review of separate analyses of one interview transcript revealed that the analysts were highly similar in their interpretations. Moreover, the analysts had continuous discussions during the course of the analysis and all remaining authors scrutinized the analyses in relation to the interviews. These aspects implicate that the data was approached from several perspectives, and thus, that the interviews were subjected to analyst triangulation. We argue that our method of using several researchers that analyzed and scrutinized the data led to a deep and nuanced understanding. Nevertheless, all qualitative analyses involve a personal interpretation of text-based material [40], and the findings need to be considered with this in mind.

### Conclusions

Parents of children treated for cancer experience psychological, existential, physical, and social struggles. During treatment, parents find themselves in an emotionally unstable situation, and focus their attention on caring for and protecting their child, resulting in neglect of their own psychological needs. After the curative treatment, parents experience challenges when trying to transition back to life as it was before the diagnosis, and experience a shift in focus from caring for their child to dealing with own emotional scars and fears. The findings suggest a need for future research that investigates satisfaction with information from healthcare professionals, how to support parents to be able to successfully return to work after the discharge from the hospital, and PTG among parents of children who transition to survivorship following a curative cancer treatment. Our findings illustrate the importance of adequate clinical routines that aim to screen for psychological distress among parents who have children diagnosed with childhood cancer, so that parents in need of psychological support may be identified and successfully treated. The findings highlight various aspects of psychological distress that such support should address, which may aid in the development of relevant interventions.
